# Dynamic Compressive Behaviors of Two-Layer Graded Aluminum Foams under Blast Loading

**DOI:** 10.3390/ma12091445

**Published:** 2019-05-03

**Authors:** Minzu Liang, Xiangyu Li, Yuliang Lin, Kefan Zhang, Fangyun Lu

**Affiliations:** 1 College of Liberal Arts and Sciences, National University of Defense Technology, Changsha 410073, China; xiangyulee@nudt.edu.cn (X.L.); yulianglin@nudt.edu.cn (Y.L.); fly3away@163.com (K.Z.); fylu@nudt.edu.cn (F.L.); 2 State Key Laboratory of Explosion Science and Technology, Beijing Institute of Technology, Beijing 100081, China

**Keywords:** aluminum foam, gradient, dynamic behavior, energy absorption, Voronoi technique

## Abstract

Experimental and numerical analyses were carried out to reveal the behaviors of two-layer graded aluminum foam materials for their dynamic compaction under blast loading. Blast experiments were conducted to investigate the deformation and densification wave formation of two-layer graded foams with positive and negative gradients. The shape of the stress waveform changed during the propagation process, and the time of edge rising was extended. Finite element models of two-layer graded aluminum foam were developed using the periodic Voronoi technique. Numerical analysis was performed to simulate deformation, energy absorption, and transmitted impulse of the two-layer graded aluminum foams by the software ABAQUS/Explicit. The deformation patterns were presented to provide insights into the influences of the foam gradient on compaction wave mechanisms. Results showed that the densification wave occurred at the blast end and then gradually propagated to the distal end for the positive gradient; however, compaction waves simultaneously formed in both layers and propagated to the distal end in the same direction for the negative gradient. The energy absorption and impulse transfer were examined to capture the effect of the blast pressure and the material gradient. The greater the foam gradient, the more energy dissipated and the more impulse transmitted. The absorbed energy and transferred impulse are conflicting objectives for the blast resistance capability of aluminum foam materials with different gradient distributions. The results could help in understanding the performance and mechanisms of two-layer graded aluminum foam materials under blast loading and provide a guideline for effective design of energy-absorbing materials and structures.

## 1. Introduction

Metal foam is a new class of ultra-light multi-functional material with the ability to undergo large deformation at a nearly constant plateau stress; and thus can absorb a large amount of kinetic energy before collapsing to a more stable configuration [1,2,3]. Foam materials exhibit three universal deformation characteristics, namely, initial linear elastic stage, extended plateau stage, and final densification stage [4,5,6]. Numerous studies and researches on the mechanical properties of metal foams mainly focused on quasi-static properties, such as tension, compression, and flexural properties, as well as their associated fracture behaviors. In order to use metal foams in advanced applications, such as crash or impact protection, blast resistance, and aeronautical and space structures, understanding their behaviors under high-rate loading is crucial [7,8,9].

Over the last decade, the dynamic behaviors of metal foams under blast loading received great interest [10,11,12]. Guruprasad and Mukherjee [9,13] studied the dynamic behaviors of a sacrificial layer under blast loading by experimental and analytical methods. Accordingly, layered sacrificial foams were observed as highly effective for energy absorption, with predictable behaviors under blast loading. Hanssen et al. [14] proposed an analytical solution that was based on one-dimensional shock-wave theory to explain the phenomenon in which the filling of a metal foam plate in tests increased the energy and impulse transfer to a pendulum. Li and Meng [15] identified dimensionless numbers of material properties and loading parameters when a foam layer was subjected to a blast pulse using a one-dimensional mass-spring model. Karagiozova and Alves [16] conducted analytical and numerical investigations to reveal the importance of cellular material topology under dynamic loading. Merrett et al. [17] reported results from experiments on aluminum foams under blast loading, and they found that little increase was exhibited in the stress during the forward direction tests. Wang et al. [18] performed investigations on the strain-rate effect and inertia effect of aluminum foam under dynamic loading. The group believed that axial-inertia effect became more important than the strain-rate effect under impact loading. Sandwich structures with graded foam cores show great potential for blast/shock resistance compared with their single counterparts. The mechanical properties of graded foam materials can be easily designed and controlled. The continuously graded foam is approximately substituted with stepwise graded foam [19,20]; however, stepwise graded foam under dynamic compression presents a more complex response in compassion with the continuously graded foam [21]. Design optimization of graded foams is meaningful and essential to avoid stress enhancement and improve energy absorption without increasing working size. Graded foams have recently received increasing attention for achieving excellent compressive response and energy absorption [22]. Metal foams with property gradient are always designed to achieve high energy absorption efficiency, thus improving the overall blast resistance of protective structures [23]. The foam gradient is due to the distribution of yield stress variation. In general, the property gradient of metal foams is achieved with density distribution gradient in industrial manufacturing.

Scholars have recently increasingly studied layer-by-layer graded materials to improve their impact/blast capability [19,24,25,26,27]. Ma and Ye [28] investigated the energy absorption of a double-layer cellular material under air blast loading based on the rigid, perfectly plastic, locking (R-PP-L) material model. The result showed that a double-layer cellular cladding could resist a much higher blast impulse. Wang et al. [29] performed shock tube experiments to study the dynamic response of sandwich panels with composite face sheets and stepwise graded styrene foam cores. They found that the total energy difference between incident and reflected energies was almost identical. Karagiozova and Alves [30] proposed a theoretical model for the propagation of stress waves in foam materials with non-uniformity to deepen the understanding of their dynamic compaction under impact loading. Liao et al. [31] proposed a strain field calculation method based on an optimal local deformation gradient technique to calculate the local strain of foam materials. The results for the mechanisms of shock front propagation provided useful understanding of double-layer foam cores. Cao et al. [32] studied the influence of the property gradient on the impact behavior of graded foams. A notable difference was observed between different property gradient profiles at high impact velocity. Liang et al. [33] developed an analytical model for the design of metal foams with continuous-density graded metal foam subjected to air-blast loading and discussed the effects of parameters on energy absorption. Chen et al. [34] investigated the design optimization of clamped sandwich panels comprising two aluminum alloy face sheets and a layered-gradient aluminum foam core under air-blast loading. The optimization results showed the trade-off relationships among maximum energy absorption, minimum structural mass, and minimum deflection. However, to the authors’ knowledge, there are few studies on the blast experiments of two-layer graded foam with no clamping considering gradient, stress wave propagation, and plastic densification wave; although this issue is much desired in the applications of foam-cored structure design stated earlier.

In summary, for graded metal foams under blast loading, the experimental result of compaction wave propagation is lacking. Furthermore, the relationship between energy absorption and impulse transmit has not been studied for blast resistance purpose. Therefore, the purpose of this paper is to demonstrate dynamic response, energy absorption, and impulse transmission of a two-layer graded foam using a combination of experiments and simulations. The results will help to understand the performance and the mechanisms of the two-layer graded foam with no clamping under blast loading and provide a guideline for a better blast-resistant structure design. The quasi-static and blast experiments were first performed to investigate the deformation and densification wave formation of the two-layer graded foams with positive and negative gradients. The stress wave pressure curves and real deformation images were carefully analyzed to reveal the densification mechanisms of these two-layer foam composites. Finite element (FE) models of two-layer graded aluminum foam were developed using the Voronoi technique. Numerical analysis was performed to simulate deformation and densification process by the software ABAQUS/Explicit 6.9. The deformation patterns were presented to provide insights into the influences of the foam gradient on compaction wave mechanisms. Based on experimental and numerical results, the absorbed energy and transferred impulse were examined to capture the effect of blast pressure and material gradient.

## 2. Experiments

### 2.1. Material Properties

**Figure 1:**
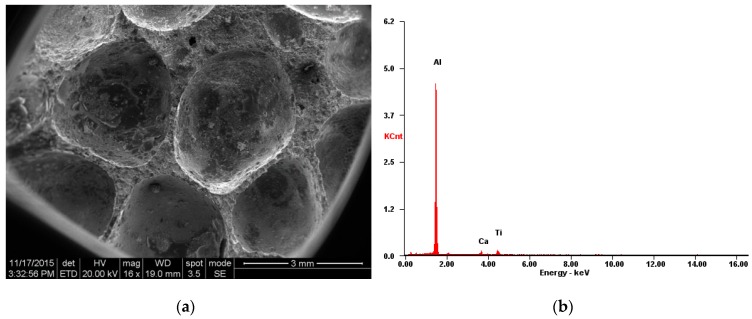
Electron microscope photographs of aluminum foam microstructures: (**a**) Electron microscope photograph; (**b**) energy dispersive spectrometer (EDS) result.

**Figure 2:**
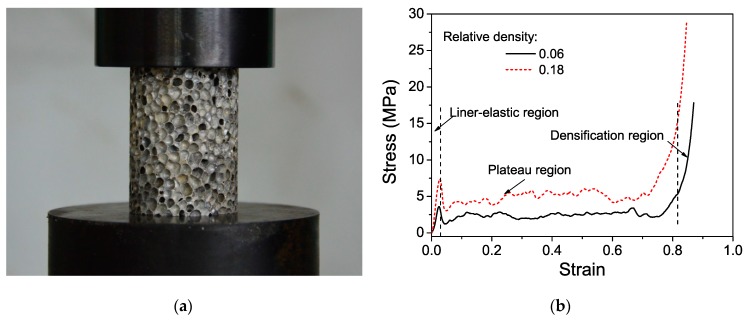
Quasi-static compression stress–strain curves of aluminum foam materials at a strain rate of 0.0005 s^−1^ (ram speed of 1 mm/min). (**a**) Specimen; (**b**) stress–strain curves.

The aluminum foam material used in this study was closed-cell foam. Figure 1a shows the typical electron microscope photograph of the aluminum foam microstructures. The aluminum foam material was made by stirring a foaming agent (TiH_2_) into an aluminum alloy and controlling the pressure while cooling. The average cell size of the material was approximately 2 mm. The energy dispersive spectrometer (EDS) result (Figure 1b) showed that the main chemical composition of the foam was 88.76% Al–3.60% Ca–7.64% Ti (by wt.%). The stress–strain curves were determined from the recorded load-displacement data using standard procedure. The cylindrical specimens were 30 mm thick and 18 mm in diameter for quasi-static testing. The tests were performed under a displacement-controlled condition at a speed of 1 mm/min. As shown in Figure 2a, sufficient cells existed in all directions, thereby effectively representing the material properties of the specimen. At least three specimens from each set were tested to check for repeatability. Stress was calculated by dividing the load by the cross-sectional area. The quasi-static compression stress–strain curves are shown in Figure 2b. The compressive deformation process of the aluminum foams, which was similar to the typical behavior of other metal foams, exhibited three universal deformation characteristics: Initial linear elastic region, extended plateau region, and final densification [26]. Plateau stress and densification strain are important parameters that characterize the mechanical properties of foam materials and have been extensively used in design and analyses. Thus, precise and clearly defined methods should be used to determine these parameters. In the current work, plateau stress and densification strain were obtained based on the ISO 13314: 2011 [35], which is the standard for porous and cellular metals. The mechanical properties of the aluminum foams are listed in Table 1. 

### 2.2. Experimental Setup

**Figure 3:**
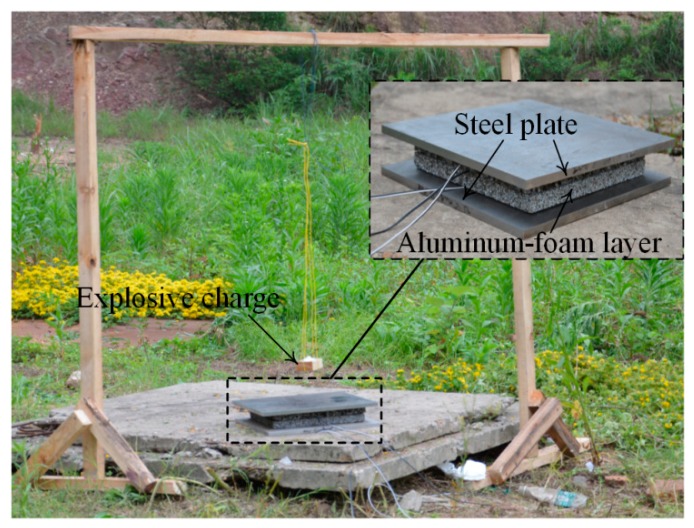
Sketch of the experimental setup.

A sketch of the overall experimental setup, consisting of explosive, layered foams, and steel plates, is shown in Figure 3. Two-layer graded foam is a material with several foam layers, whose density distributes layer-by-layer in space. The specimen used in this paper was two-layer graded aluminum foam. The aluminum foam specimen was placed between two steel plates. The specimen consisted of two foam layers with different densities, and the relative densities of the soft and hard foams were 0.06 and 0.18, respectively. A foam layer was a square of 120 mm and thickness of 20 mm and was cut to shape with an electro-discharge machine to minimize damage to the cell edges. Then, the aluminum foams were annealed at 393 K for 1 h to relieve residual stress in the material during the manufacturing of the base material or machining process. The gradient is positive if the soft layer locates near the blast end, and the gradient is negative when the soft layer locates near the distal end. The soft and hard layers were placed at blast ends in tests 1 and 2 (the specimens were defined as positive and negative gradients), respectively. Details of the specimens and blast loadings are listed in Table 2. Layer 1 was closed to blast loading, and layer 2 was at the distal end. The high explosive with cubic shape was centrally held on the top face of the front plate at a vertical standoff distance of 200 mm and detonated at its apex with a detonator. Steel plates made from AISI 1045 steel consisted of a square of 160 mm with a 10 mm thickness. The front plate was placed on the layered foams to transmit detonation pressure.

Polyvinylidene fluoride (PVDF) piezoelectric sensors were placed between adjacent foam layers to monitor dynamic stress. PVDF is effectively applied for stress measurement under dynamic conditions because of its particularly short response time. The thickness of the PVDF sensor was only 40 μm, and the period of shock wave propagation was negligible. The instantaneous stress measured by the PVDF gauges is

(1) $$\sigma_{pvdf}=iQ/D$$

where *i* is the sensitivity coefficient of the PVDF sensor, which is calibrated by the Hopkinson pressure bar, and *i* = 40.5 N/C in this study. *Q* is the total electrical charge, and *D* is the work area of the PVDF sensor.

(2) $$Q=\int_{0}^{t}\frac{U\left(t\right)}{R_{p}}dt$$

where *U*(*t*) is the voltage measured, and *R_p_* is the resistance of the parallel connection.

### 2.3. Experimental Results

**Figure 4:**
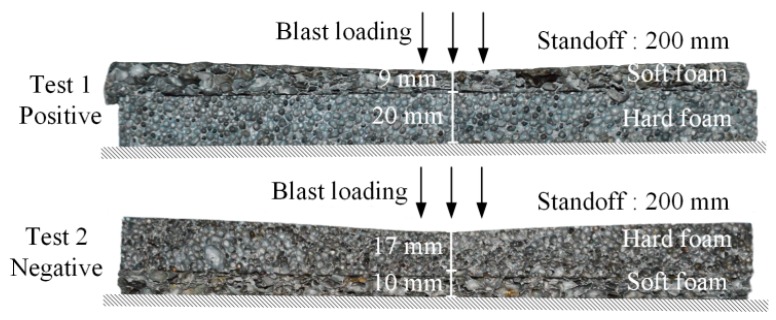
Cross-sections of deformed specimens.

**Figure 5:**
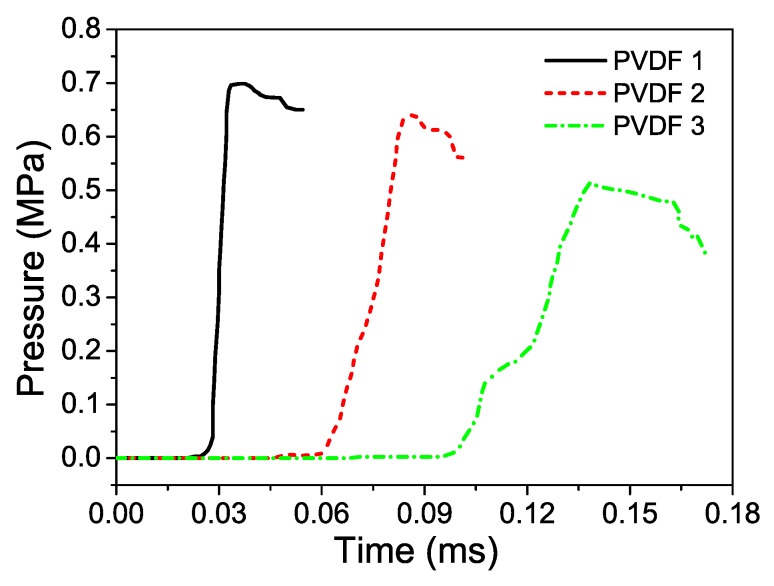
Pressure–time histories in positive aluminum foam specimen (test 1). PVDF 1 was sandwiched between an upper steel plate and layer 1; PVDF 2 was sandwiched between layer 1 and layer 2; PVDF 3 was sandwiched between layer 2 and the distal steel plate.

The cross-sections of deformed specimens are shown in Figure 4. In test 1, layer 1 collapsed by more than a half, whereas layer 2 was not deformed. This result indicated that the compaction started from the blast end and then propagated to the fixed end when the gradient was positive. In test 2, the hard and soft layers finally partly deformed when the specimen was negative. The compaction areas were all the upper parts, thereby indicating that the compaction wave started at the proximal end of each layer and propagated to the fixed end. Deformation dimensions of deformed specimens are listed in Table 3. However, the deformation process could not be obtained in the tests. The original voltage curves of the blast wave were recorded by the PVDF piezoelectric sensors in the tests. According to Equations (1) and (2), the pressure–time curves were obtained (Figure 5). Evidently, stress wave attenuation and extended rise time were observed in the propagation process. The shape of the stress waveform changed in the propagation process, and the time of edge rising was extended.

## 3. Numerical Simulation

### 3.1. Voronoi Technique

**Figure 6:**
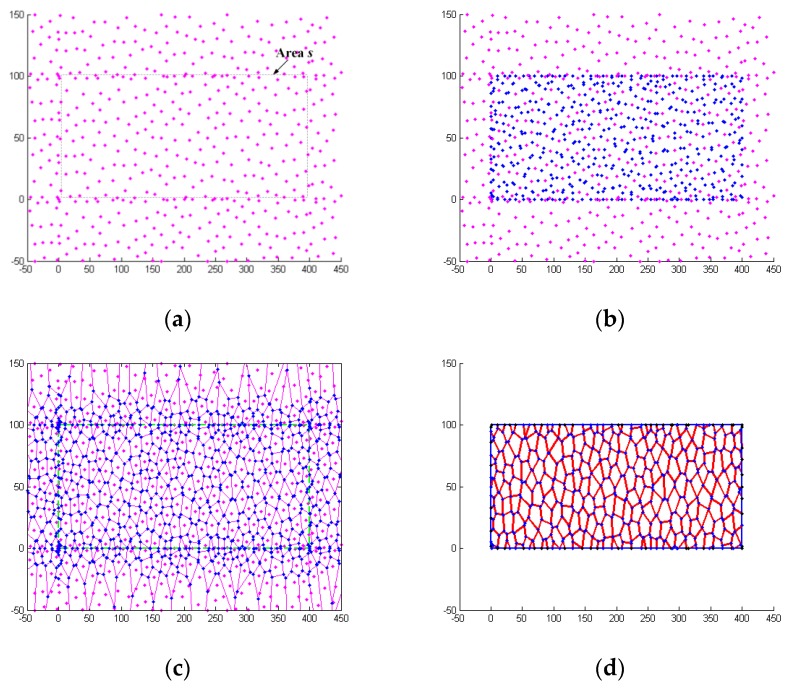
Four main stages of the Voronoi technique: (**a**) Nuclei in given space *s*; (**b**) Delaunay triangulation and Voronoi diagram; (**c**) Voronoi structure.

The Voronoi technique was used to develop an FE model of the aluminum foam material by MATLAB R2012a. This technique can be generally described in four main stages, as shown in Figure 6. At the first stage, *n* nuclei are randomly generated in a given area space, *s*, on the basis of the principle that the distance between any two nuclei is constrained to be larger than a given minimum distance, δ_min_. The geometrical relationship indicated that the average distance, δ_0_, between any two adjacent nuclei is given by

(3) $$\delta_{0}=\sqrt{2s/\sqrt{3}n}$$

The relationship between δ_min_ and δ_0_ is

(4) $$\delta_{\min}/\delta_{0}=1-k$$

where *k* is the degree of foam cell irregularity, and *k* = 0.2 is used in this study. At the second stage, the generated nuclei at the first stage are copied to the surrounding neighboring regions by the translation operation. Then, the Delaunay triangulation and Voronoi diagram are developed. For a Voronoi diagram, a Voronoi cell contains all points that are closer to its data point than any other data points in the set. Finally, out of the given area, *s* is deleted, and the geometry of the foam material is obtained. The relative density of the foam is defined as

(5) $$\rho =\rho_{f}/\rho_{m}=\sum h_{j}l_{j}/s$$

where ρ*_f_* is the density of the foam, ρ*_m_* is the density of the base material of the foam, *l_j_* is the cell wall length, and *h_j_* is the corresponding wall thickness.

### 3.2. FE Model

**Figure 7:**
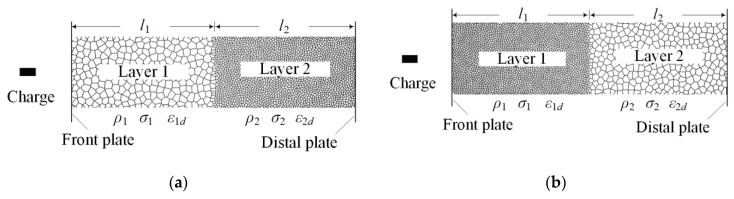
Finite element (FE) model of aluminum foam subjected to TNT charge. (**a**) Positive gradient specimen, *g* > 0; (**b**) negative gradient specimen, *g* < 0.

The Voronoi technique was used to generate an FE model of the aluminum foam. Numerical simulations were performed by ABAQUS/Explicit [36]. As shown in Figure 7, the aluminum foam material was placed between two steel plates, and the plate at the distal end was fixed. The foam material was created in an area of 100 mm × 400 mm. The two-layer graded foam was divided into two equal-sized layers with 200 mm height. The base material of the aluminum cell wall was assumed as an elastic–perfectly plastic model. The parameters of the base material are listed in Table 4.

The explosive charge was held at a horizontal standoff distance relative to the proximal end. The Jones–Wilkins–Lee (JWL) model was used for the charge products, and the pressure of the detonation products was given as a function of relative volume and internal energy per initial volume as follows:

(6) $$p_{e}=A\left(1-\frac{{\omega \rho }_{e}}{R_{1}\rho_{e0}}\right)e^{-R_{1}\frac{\rho_{e}}{\rho_{e0}}}+B\left(1-\frac{{\omega \rho }_{e}}{R_{2}\rho_{e0}}\right)e^{-R_{2}\frac{\rho_{e}}{\rho_{e0}}}+\frac{{\omega \rho }_{e}}{\rho_{e0}}E_{m0}$$

where *p_e_* is the detonation pressure; ρ*_e_* is the explosive density; *ρ_e_*_0_ is the initial density of detonation products; *A*, *B*, *R*_1_, *R*_2_, and ω are material constants; and *E*_m0_ is the detonation energy density. The JWL parameters of the TNT charge are listed in Table 5.

The foam cell was meshed using the ABAQUS shell element S4R [37]. Self-contact was applied to all the cell surfaces. Meanwhile, general contact was considered between the Voronoi structure and the front plates with a friction coefficient of 0.02 [37,38]. The density gradient of an aluminum specimen, *g*, was defined as

(7) $$g=\frac{\sigma_{2}l_{2}-\sigma_{1}l_{1}}{\left(\sigma_{1}+\sigma_{2}\right)\left(l_{1}+l_{2}\right)},$$

where *l*_1_ and *l*_2_ are the thicknesses of layers 1 and 2, and corresponding plateau stresses are σ_1_ and σ_2_, respectively. The gradient is positive if the soft layer locates near the blast end. The gradient of homogeneous foam material is zero. When the number of foam specimen is more than two, the gradient could be defined by the case of adjacent layers.

## 4. Results and Discussion

**Figure 8:**
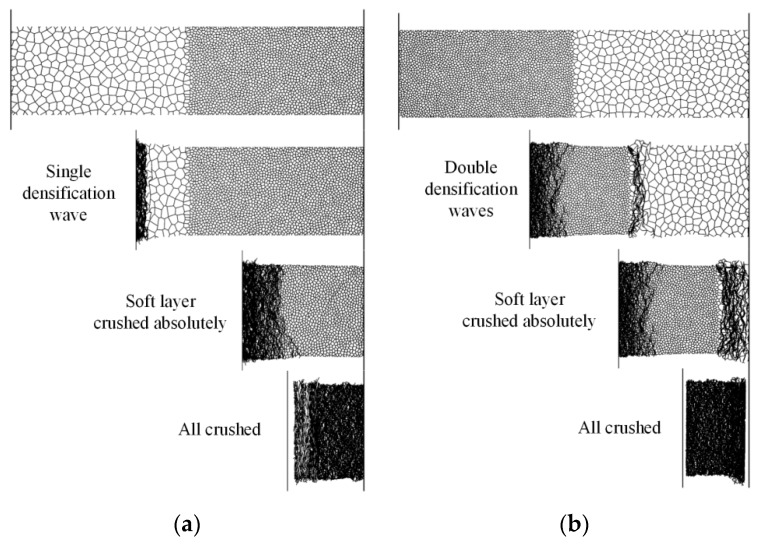
Deformation processes induced from FE simulations. (**a**) Positive gradient specimen, *g* = 0.3; (**b**) negative gradient specimen, *g* = −0.3.

The simulation results of positive and negative distributions are plotted in Figure 8. The deformation was highly localized, and progressive cell crushing was observed to propagate like a shock wave through the aluminum foam material under blast loading. The shock wave refers to the shock-like compaction wave with a fast propagating thin area termed as a shock-front, separating the deformed and undeformed regions. Table 6 compares the average cross-section thicknesses of deformed specimens. A good agreement was achieved between experimental results and FE predictions. The tests validate the FE results are reasonable.

**Figure 9:**
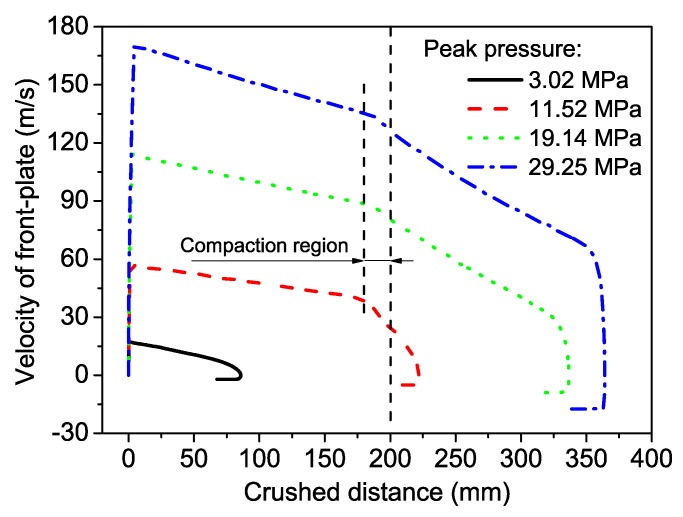
Velocity and crushed distance of the front plate for a positive gradient foam. *g* = 0.3.

When the gradient was positive, only one shock wave propagated from the blast to the fixed end during the complete crushing process. This phenomenon coincided with uniform foam. When the gradient was negative, the hard layer was placed at the blast end. Densification was first observed in layer 1 at the blast end. Subsequently compaction started in layer 2 at the end near layer 1 when the stress wave reached layer 2. However, the double shock fronts were in the same direction, which was different from the continuous gradient foam that had double shock fronts with opposite directions [40]. Layer 2 crushed faster than layer 1 due to its low plateau stress. Therefore, layer 2 first completely compacted, followed by layer 1. However, compaction velocity depended on the plateau stress of layers 1 and 2. Hence, two direct phases emerged at the deformation process for a double-layer foam material. This phenomenon was also observed in the velocity curves of the front plate, as illustrated in Figure 9. The crushed distance of the front plate did not reach 200 mm when layer 1 was completely compacted due to the presence of a certain thickness of the crushing zone behind the front plate. In addition, the maximum crushed distance and velocity increased with increasing blast loading.

Determining the absorbed energy and transmitted impulse of the two-layer graded aluminum foam under blast loading is of practical interest. Aluminum foam material with high energy absorption and low transmitted impulse to protected structure is an excellent choice for securing the structure because it could meet crashworthiness requirements. Elastic deformation energy, unlike plastic deformation energy, could be neglected. The densification strain can be regarded as a constant under blast loading, given that the dynamic load had a slight influence on the densification strain, ε*_d_* [41]. The densification strain varied with relative densities of foam materials. The plastic deformation energy absorption of two-layer graded aluminum foam under quasi-static *E_q_* can be obtained by

(8) $$E_{q}=l_{1}\sigma_{1}\varepsilon_{1d}+l_{2}\sigma_{2}\varepsilon_{2d}$$

where ε_1*d*_ and ε_2*d*_ are the densification strains of layers 1 and 2, respectively. The energy absorption of two-layer graded aluminum foams subjected to dynamic loading, *E_d_*, can be obtained by

(9) $$E_{d}=\int_{0}^{l_{1}}\frac{1}{2}\left(\sigma_{1}+\sigma_{1d}\right)\varepsilon_{1d}d\xi +\int_{0}^{l_{2}}\frac{1}{2}\left(\sigma_{2}+\sigma_{2d}\right)\varepsilon_{2d}d\xi$$

The R-PP-L material model was first studied by Reid and Peng [42] to explain the crushing enhancement of wood specimens. This theory was successfully applied for various metal foams subjected to dynamic loading. The crushing stress, σ*_d_*, as a function of the velocity of the compaction wave front, *v*, was presented through preserving mass and momentum at the shock front and idealizing a foam material as the R-PP-L [43]

(10) $$\sigma_{d}=\sigma_{0}+\rho v^{2}/\varepsilon_{d}$$

where σ_0_ and ε_d_ are the plateau stress and densification strain, respectively.

The energy absorption of two-layer graded aluminum foam can be rewritten by substituting Equations (10) and (8) into Equation (9) as follows:

(11) $$E_{d}=E_{q}+\frac{1}{2}\left(\int_{0}^{l_{1}}\rho_{1}v{\left(\xi \right)}^{2}d\xi +\int_{0}^{l_{2}}\rho_{2}v{\left(\xi \right)}^{2}d\xi \right).$$

**Figure 10:**
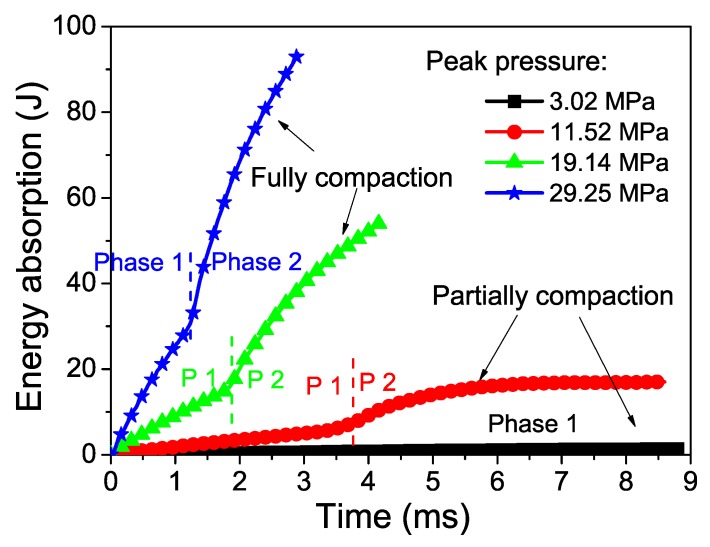
Energy absorption of two-layer graded foam under different blast pressures. *g* = 0.3.

The abovementioned equation indicates that the energy absorption could be calculated (Figure 10). The energy absorption capacity increases with blast pressure due to high dynamic plateau stress and crushed displacement under higher blast pressure. As shown in Equation (10), high velocity leads to high crushing stress. Two phases for energy absorption also exists, even when the blast pressure is relatively small.

**Figure 11:**
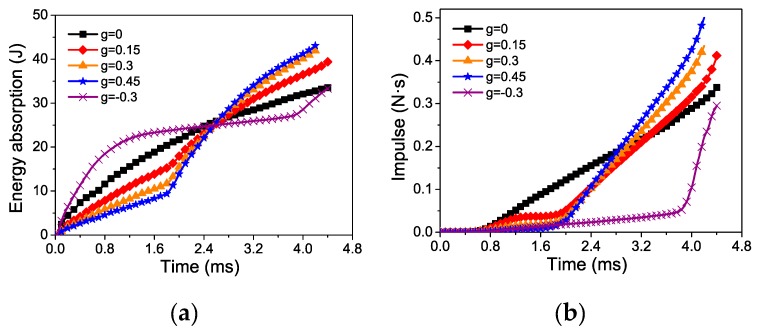
Gradient effects on two-layer graded aluminum foam under blast loading: (**a**) Energy absorbed; (**b**) impulse transmitted.

The impulse transmitted to the fixed end, *I_d_*, is obtained by

(12) $$I_{d}=\int f_{fixed}dt$$

where *f*_fixed_ is the force transmitted to the protected structure. The gradient effect on energy absorption and transmitted impulse of the two-layer graded foam is shown in Figure 11. Evidently, the greater the foam gradient, the higher was the energy absorption. The aluminum foam with *g* = 0 (uniform density) had an energy absorption of 33.5 J, whereas those with *g* = 0.15, 0.3, and 0.45 had an energy absorption of 39.4, 41.7, and 43.1 J, i.e., a 17.6%, 24.4%, and 28.7% increase, respectively. The energy absorption of foam material with *g* = −0.3 was lower than that of the corresponding single counterpart, which indicates the negative gradient has no advantage in energy absorption. With a negative density gradient, the impulse transmission and energy absorption were all the lowest. The aluminum foam with *g* = −3 had an impulse transmission of 0.295 Ns, whereas those with *g* = 0.15, 0.3, and 0.45 had an impulse transmission of 0.412, 0.434, and 0.499 Ns, i.e., a 39.7%, 47.1%, and 69.2% increase, respectively. Therefore, the two-layer graded aluminum foam specimens with high energy absorption transmitted substantial impulse to the protected structure. The energy absorption capability and transferred impulse to the fixed end were conflicting objectives in the evaluation of protective capability.

## 5. Conclusions

Dynamic behaviors of two-layer graded aluminum foam materials subjected to blast loading were analyzed using experiments and numerical simulations. Experiments were performed to investigate the deformation behaviors and stress wave propagation. Stress wave attenuation and extended rise time were observed using the PVDF sensor. The results indicated that the shape of the stress waveform changed during the propagation process, and the time of edge rising was extended. The compaction areas in the tests were all the upper parts, thereby indicating that the compaction wave started at the proximal end of each layer and propagated to the fixed end. FE models were developed using the Voronoi technique, and numerical simulations were conducted by ABAQUS to capture densification wave compaction, energy absorption, and transmitted impulse. The deformation patterns of two-layer graded foams were presented to provide insights into the influence of foam gradient on compaction wave mechanisms. When the gradient was positive, only one shock wave propagated from the blast to the fixed end during complete crushing process. This phenomenon coincided with uniform foam. When the gradient was negative, double shock fronts were in the same direction, which was different from continuous graded foam that had double shock fronts with opposite directions.

The energy absorption and transferred impulse were examined to capture the effect of blast pressure and material gradient. The energy absorption capacity of the two-layer graded foam increased with blast pressure due to high dynamic plateau stress and crushed displacement under enhanced blast pressure. The greater the foam gradient, the more energy dissipated and the more impulse transmitted. With a negative density gradient, the impulse transmission and energy absorption were all the lowest. It is indicated that the graded foams with high energy absorption transmit substantial impulse to the protected structure. The aluminum foam with high energy absorption and low transmitted impulse to the protected structure is thus an excellent choice for securing the structure because it could meet crashworthiness requirements. This finding indicated that absorbed energy and transferred impulse are conflicting objectives for the blast resistance capability of aluminum foam materials with different gradient distributions. These results could help in understanding the performance and mechanisms of graded aluminum foam materials under blast loading and provide a guideline for the effective design of energy-absorbing materials and structures.

## Figures and Tables

**Table 1:** Mechanical properties of the aluminum foams.

Relative Density ^*^	Yield Stress (MPa)	Standard Deviation (MPa)	Plateau Stress (MPa)	Standard Deviation (MPa)	Densification Strain	Standard Deviation
0.06	3.59	0.22	2.51	0.15	0.77	0.12
0.18	7.29	0.38	5.07	0.24	0.72	0.11

^*^ The density of bulk material is 2.78 g/cm^3^.

**Table 2:** Details of specimens and blast loads.

Test No.	TNT Charge (g)	Standoff Distance (mm)	Number of Layers	Specimen Gradient	Specimen Thickness (mm)	
					Layer 1	Layer 2
1	100	200	2	Positive	20	20
2	100	200	2	Negative	20	20

**Table 3:** Deformation dimensions of deformed specimens.

Test No.	Specimen Gradient	Initial Thickness/(mm)		Thickness of Deformed Specimen/(mm)		Deformation/(mm)	
		Layer 1	Layer 2	Layer 1	Layer 2	Layer 1	Layer 2
1	Positive	20	20	9	20	11	0
2	Negative	20	20	17	10	3	10

**Table 4:** Parameters of the base material of aluminum foam [33].

Base Material	Density/(kg/m^3^)	Young Modulus/(GPa)	Poisson Ratio	Yield Stress/(MPa)
Aluminum alloy	2780	70	0.3	190

**Table 5:** Jones–Wilkins–Lee (JWL) model parameters of the TNT charge [39].

Charge	*A* (Mbar)	*B* (Mbar)	ω	*R*_1_	*R*_2_	*E*_m0_ (J/mm^3^)
TNT	3.74	0.032	0.3	4.15	0.95	70

**Table 6:** Comparisons of average cross-section thicknesses of deformed specimens.

Results	Positive Foam/(mm)		Negative Foam/(mm)	
	Layer 1	Layer 2	Layer 1	Layer 2
Experimental results	9	20	17	10
Simulation predictions	9.2	20	17.1	9.9
